# 

**Published:** 1917-04-14

**Authors:** 


### Out-Patient Treatment of Discharged Soldiers.

The arrangements in respect of hospital treatment and training for civil employment of discharged soldiers has now been communicated to the military hospitals, and voluntary hospitals taking soldiers, on an Army Council instruction and a poster. The latter informs military patients that when men still require to continue the hospital treatment which was being applied at the moment of their discharge they can be given this treatment in hospitals near their homes. This treatment is to be arranged for the patient by the local War Pensions Committee, and apparently, from the wording of the Army Council instruction, it will be afforded at a military hospital, for provision is made to enable the D.D.M.S. of a Command to supply a committee with "lists of military hospitals which will serve the various districts concerned" and of "special hospitals in the Command." Does this mean that the services of the voluntary hospitals in treating discharged soldiers will not be called upon at all? In respect of training it is provided that any man who suffers from any disability of a permanent nature unfitting him for his ordinary occupation can be taught, if he so desires, work in which there is a good prospect of his obtaining profitable employment, and can be provided with practical advice in connection with such employment.

Rates of Subscription: Twelve months, 6/6; Six months, 4/-; Three months, 2/-; post free to any address in the United Kingdom. Abroad, Twelve months, 8/8; Six months, 5/-; Three months, 2/6, post free. The Hospital "Index" to Volume LX., April to September 1916, is now ready, price 2½d. per copy, post free. Address The Manager, "The Hospital," The Hospital Building, 28/29 Southampton Street, Strand, London, W.C. 2.

28/29 Southampton Street, Strand, London, W.C. 2.

### HUMAN TEMPERAMENTS:

### Studies in Character.

By Dr. CHARLES MERCIER.

**1/3 net. By Post 1/4.**

"The essays are written in Dr. Mercier's well-known style—they are witty, pithy, and display a wide range of observation and learning. We recommend the brochure cordially to our readers. It is always extremely readable and often very wise."—*The Lancet.*

"Dr. Mercier is a well-known authority on mental diseases, and his practical knowledge assists him in this analysis of the temperaments of the artist, the faddist, the practical man, the envious man, the philosopher, and so on. Dr. Mercier can claim that the reader may find some widening of his knowledge of human nature as the result of these shrewd and experienced studies."—*The Times.*

"It is long since within so small a compass so much help was given to mankind in the pursuit of that 'proper study,' the knowledge of his fellow-man."
*The Hospital.*

*Of all Booksellers, or of*

**The Scientific Press, Ltd.,**

**28/29 Southampton Street, Strand, London, W.C. 2**

